# Use and disuse of malaria bed nets in an internally displaced persons camp in the Democratic Republic of the Congo: A mixed-methods study

**DOI:** 10.1371/journal.pone.0185290

**Published:** 2017-09-26

**Authors:** Hannah Myfanwy Brooks, Makelele Katsuva Jean Paul, Kasereka Masumbuko Claude, Victor Mocanu, Michael T. Hawkes

**Affiliations:** 1 School of Public Health, University of Alberta, Edmonton, Alberta, Canada; 2 Médecins Sans Frontières, Goma, Democratic Republic of Congo; 3 Université Catholique du Graben, Butembo, Democratic Republic of Congo; 4 Department of Sciences, University of Alberta, Edmonton, Alberta, Canada; 5 Department of Pediatrics, University of Alberta, Edmonton, Alberta, Canada; Centro de Pesquisas Rene Rachou, BRAZIL

## Abstract

**Introduction:**

Malaria is a major cause of morbidity and mortality among displaced populations in tropical zones. Bed nets are widely used to prevent malaria; however, few data are available on bed net distribution within displaced populations.

**Methods:**

Mixed methods study in a single internally displaced persons (IDP) camp and neighboring community in Eastern Democratic Republic of the Congo (DRC). Qualitative data (focus group discussions, FGDs) and quantitative data (door-to-door survey and individual testing using malaria rapid diagnostic test, RDT) were collected.

**Results:**

Ten FGDs were conducted with 55 individuals. Although malaria was widely recognized as a significant threat and bed nets were freely distributed in the camp, many households did not own or use them. IDPs converged on the following reasons for low bed net ownership and use: inconvenience of net installation and sale of nets to meet immediate needs such as food. One hundred households, comprised of 411 individuals, were surveyed in Birambizo. The burden of malaria was high (45/78 (58%) of children <5 were positive for malaria by RDT) and bed net utilization was low (29/100 (29%) households owned a bed net, and 85/411 (20%) individuals slept under a bed net the previous night). Children <5 were more likely to use a bed net than older children or adults (OR 3.4 (95%CI 2.0–5.8), p<0.0001). Compared to 29 bed nets currently in use by study participants, 146 bed nets had been sold (82%) or exchanged (18%) either in the camp (27%) or in the neighbouring village market (73%).

**Conclusions:**

Qualitative descriptions and quantitative analysis revealed pragmatic barriers to bed net usage and widespread sale of freely distributed bed nets within IDP camps, despite a high burden of malaria. Additional strategies, beyond bed net distribution, are warranted to combat malaria in vulnerable and hard-to-reach population.

## Introduction

Malaria is one of the most common causes of global childhood mortality, accounting for an estimated 429,000 deaths annually [1]. Malaria is caused by *Plasmodium* species and is transmitted by the female *Anopheles* mosquito, such that vector control measures including bed nets and indoor residual spraying with insecticides are important tools for malaria control. Cases of malaria have highest incidence in sub-Saharan Africa, where children under 5 account for 70% of malaria deaths globally [1].

Recent global estimates suggest that violent conflicts and natural disasters resulted in ~20 million refugees and ~40 million internally displaced persons (IDP) fleeing their homes in 2016 [2,3]. Whereas most refugees were from Syria, Afghanistan and Somalia, the majority of IDPs were in the Democratic Republic of the Congo (DRC), Colombia and Iraq [4–6]. Children make up more than half of the refugee population and a higher proportion of IDPs [3,7]. These mobile populations, fleeing their homes due to violent conflict, are difficult to reach and often lack stable health infrastructure; therefore, disease prevention, diagnosis, and treatment efforts may be compromised. Often living in crowded camps, these vulnerable populations generally have limited access to quality shelter, sanitation, clean water, stable food supply, and healthcare [8]. Under these conditions, malaria prevention and treatment efforts are challenging and malaria transmission is elevated [9–11]. Furthermore, few medical facilities are available to diagnose and treat individuals living in displacement camps.

Following decades of civil and international conflict, the DRC has more than 1.5 million IDPs [12]. The incidence of malaria in the DRC is estimated to be over 6 million cases annually, and malaria is the leading cause of childhood mortality in the country. We and others have previously reported that the burden of malaria is disproportionately high in Congolese IDP camps, and that bed nets are under-utilized [9,13,14]. The purpose of this study was to explore the factors influencing bed net ownership and use in an IDP camp with universal free bed net distribution using mixed qualitative and quantitative methods. We first sought to understand the reasons for the lack of bed net ownership and use in this high-risk population through focus group discussions (FGDs) with key informants, including IDPs, neighboring villagers, and health care providers working in the displacement camp. Results arising from thematic analysis of FGDs were then used to design and implement a door-to-door survey of a random subset of IDP households to quantify bed net use and disuse, as well as explanatory factors.

## Methods

### Study setting

This study was conducted in the IDP camp of Birambizo and the nearby villages of Kizimba, Budei and Nyangutu, in the Walikale district of North Kivu province in the DRC. Approximately 255,530 individuals live within the 1,967 km^2^ region, of which over 73,000 are IDPs. A recent investigation in this region found the leading cause of mortality in children under 5 years of age to be malaria [15].

For the past decade, the Birambizo health zone has faced political and ethnic based conflict, leading to the destruction of homes, banks, schools, medical centers, and the degradation of human security. The health zone is now effectively militarized, which adds to the tension and conflict in the region. Birambizo IDP camp is made up of a constantly evolving number of IDPs, who arrive and leave as a function of continuous conflict and in search of a safer area. At the time of our research, approximately 13,700 IDPs lived in Birambizo IDP camp. Following 4 attacks directed against humanitarian agencies in December 2014 and March 2015, all NGOs in the region have now stopped permanent activities in this area.

### Design

We used mixed qualitative (FGDs) and quantitative (cross-sectional survey) methods.

#### Focus group discussions

FGDs were conducted in Birambizo and Kizimba in April, June, and December of 2015. Key informants, including IDPs living in Birambizo IDP camp, residents from communities neighboring the displacement camp, and health care providers serving the displacement camp participated in FGDs. Overall there were 10 FGDs: 9 involved residents of the IDP camp or neighboring village, and one involved health workers (nurses). Participants were purposively sampled by the IDP camp leaders and health workers, to include bed net users and those who didn’t use a bed net, IDP camp residents and residents of neighboring village, males and females. FGDs were uniform or mixed with respect to these participant characteristics (S1 Table). FGDs were conducted in the local languages (Kiswahili, Kinyarwanda and Kinande) by members of the IDP camp with 3–6 participants in each group and lasted approximately 45 minutes each [16]. Leaders of the FGDs were briefed on the qualitative methodology by MKJP. Discussions were audio recorded, then translated and transcribed by MKJP for subsequent analysis. FGD questions were elastic, open-ended and probing, allowing participants to shape the discussion. Questions were adapted over the course of the FGDs, aiming to explore emerging themes in greater depth. FGDs were continued until a point of saturation, when no new themes emerged in discussions [17]. Thematic analysis was used to identify, analyze and report themes in the data [18]. Two investigators (VM and HJ) read the transcripts several times and noted preliminary ideas, producing initial codes by highlighting relevant data. They then generated themes by collating codes across the data set, and refining themes. Representative quotations as well as statements of particular interest were extracted and organized according to theme to support the qualitative findings. Themes that emerged from the FGDs were used to inform quantitative survey content.

#### Cross-sectional survey

Major themes from FGDs were incorporated with questions from a standardized malaria indicator survey toolkit [19] to create a novel 50 item questionnaire for the quantification of barriers to bed net ownership and use among IDPs (S2 and S3 Tables). Community health workers, trained in malaria rapid diagnostic testing as previously described [20], visited a random sample of 100 family units (“households”) within the Birambizo IDP camp. Random sampling of temporary households in the camp was performed using a census created by NGOs providing services in the area. All households on the census were eligible for inclusion. Testing for *P*. *falciparum* infection using a HRP2-based rapid diagnostic test (RDT) (Paracheck-Pf^®^) was offered to all household residents. All participants who tested positive for *P*. *falciparum* were treated with the artemisinin-based combination therapy according to WHO recommendations [21]. Heads of households were asked questions relating to household composition, demographics, bed net ownership and use (for the complete dataset refer to S1 Dataset).

### Statistical analysis

A standard sample size calculation indicated that 95 households would be needed to estimate the proportion of households that own a bed net to within ±10%, with 95% confidence, assuming that the proportion of bed net ownership was 34%, based on previous studies in the area [9]. For descriptive statistics, binomial 95% confidence intervals were calculated for proportions. Comparative statistics were computed using non-parametric methods (Mann-Whitney U-test) for continuous variables and Chi squared or Fisher’s exact test for dichotomous variables, as appropriate. Correlations between continuous variables were analysed by the non-parametric Spearman’s rank correlation coefficient. Household wealth was approximated as follows: ownership of household assets or household construction characteristics were coded as binary variables and weighted using principal component analysis (PCA). This method was modified from Filmer and Pritchett [22], and has been validated as a measure of household consumption and poverty, and has been used in previous studies in sub-Saharan Africa [23]. Eleven assets or household characteristics from the questionnaire were included to determine a wealth index; this index was then used to divide the cohort into households above and below the median wealth index. Measures that we included were: household characteristics (electricity, tarpaulin vs mud wall construction) and asset ownership (bicycle, motor vehicle, radio, telephone, television, refrigerator, chicken, cow and goat). Statistical software SPPS version 19 (IBM, USA) was used for the analysis.

### Ethics approvals and permissions

All participants provided written informed consent. Ethics approval was obtained from Comité d’Éthique du Nord Kivu (Université Catholique du Graben, ref 002/TEN/2012), the University of Alberta Human Research Ethics Board (ref Pro00055619), and regionally from the Médecin Chef de Zone, within the DRC Ministry of Health.

## Results

### Focus group discussions

We explored several potential barriers to the use of bed nets for malaria prevention through FGDs, which were grouped thematically: (1) awareness of malaria as a cause of morbidity and mortality; (2) knowledge of bed nets as an effective malaria preventative measure; (3) access and availability of bed nets; (4) pragmatic barriers to bed net use; and (5) competing needs that compel IDPs to trade or sell the bed net. Here we present each theme, together with representative quotations, translated into English from the language of the FGD.

#### (1) Awareness of malaria as a cause of morbidity and mortality

Participants were generally aware that malaria is a major cause of mortality among young children. One participant recounted his personal loss related to malaria: ‘*I take [malaria] seriously because I recently lost my eldest son of three years [of age] to this vile disease’ (IDP)*. Nonetheless, gaps in knowledge about malaria were noted by health care providers in the IDP camp. One FGD participant, a health care worker in the IDP camp, felt that self-treatment and/or the use of traditional herbal remedies was a factor contributing to severe malaria in the camp: ‘*We live in a rural area where the level of knowledge remains [primitive]’ (Nurse)*. Another nurse gave the following opinion: ‘*Many of the cases we see are complications of uncomplicated malaria cases that were [mismanaged] at home’ (Nurse)*.

#### (2) Knowledge of bed nets as an effective malaria preventative measure

Participants were widely aware that bed nets prevent malaria: ‘*Bed nets are effective because the displaced people who use them don’t often fall ill’ (IDP)*. Some cited positive personal experience with bed nets: ‘*Because I use a bed net regularly*, *I rarely fall ill*, *and even my baby*, *compared to the neighbour’s baby who doesn’t use a bed net’ (IDP)*. Others went beyond free distribution programs to purchase bed nets for their family: *‘My wife had the chance to receive a bed net when she was pregnant and we still use it*. *I bought two more bed nets for our children’ (IDP)*. FGD participants from outside the IDP camp also recognized the value of bed nets: *‘The effectiveness of bed nets was proven in our village because without using the bed net*, *we risk getting sick every month or even two to three times per year’ (Villager)*. Health information on bed nets was routinely provided in the camp, as noted by several respondents: ‘*Information about the bed nets is always given at the health centre to pregnant women*, *but there are those who don’t receive the information about the usefulness of bed nets in malaria prevention*, *especially certain pregnant women who don’t attend their antenatal visits’ (IDP)*.FGD participants trusted this information provided by health workers: ‘*The nurse from the health centre was telling the truth during the antenatal visit about the protection bed nets give against illness’ (IDP)*.

#### (3) Access and availability of bed nets

FGD participants generally had access to bed nets through free distribution programs, sponsored through the DRC government health services as well as NGOs: ‘*NGOs ensure the distribution of blankets*, *tarps*, *bed nets and other items for the IDPs’ (Villager)*. One mechanism of bed net distribution was through antenatal care (ANC) visits: ‘*We all*, *IPD or not*, *receive a bed net during antenatal visits to protect us against malaria’ (Villager)*. Nonetheless, gaps in the distribution programs were reported by some participants: ‘*I didn’t get the chance to benefit from a bed net because the quantity of nets distributed was not sufficient’ (IDP)*.

#### (4) Pragmatic barriers to bed net use

Given that malaria awareness was high, bed nets were perceived as useful, and were available free of charge, why did IDPs not use them more frequently? Uniquely challenging living conditions in the IDP camp may compromise the utility and effectiveness of bed nets. FGD participants noted pragmatic limitations, such as size of the dwelling and the mud floor: ‘*The state of our tents doesn’t encourage the use of bed nets because the tent is small and the net falls on the floor*, *so each time we have to wash it but lack of soap is an issue’ (IDP)*. Space constraints in the tent meant that bed nets required daily installation at dusk and dismantling at dawn. The bed nets were easily soiled in the muddy environment, required frequent washing, tore easily and developed holes, resulting in short life spans: *‘We must clean the nets at least once a week [which] damages it, we also don’t have permanent water sources in the camp, and soap is an issue’ (IDP); ‘With the candle that lights our night, my bed net now has many burn holes because the tent is* [24] *small to separate the net from the candle’ (IDP)*. IDPs usually slept on mats on the mud floor, such that bed nets could not be tucked under a mattress to provide a physical barrier against mosquitoes: *‘It’s difficult to use the nets well […] We have no beds or mattresses to tuck the nets under*, *we have no bedrooms’ (IDP)*. Sleeping conditions for children under five (a target high-risk group for malaria prevention) are often cramped; children often slept between parents in tents intended for two users and a second tent was often required for additional family members. One FGD participant summarized the impracticalities of bed nets in the IDP camp as follows: ‘*NGOs should think more about food before any bed net distribution because the bed nets that [they] give out aren’t even suited to our tents’ (IDP)*.

Other factors were not specific to the IDP camp, but nonetheless influenced bed net acceptability. Some complained of the chemical smell: ‘*The bed nets give off a nauseating smell that disrupts our sleep and affects our breathing’ (IDP)*. Others expressed anxieties about adverse health effects: *‘Some say the nets are toxic when they give off odour’ (IDP)*.

#### (5) Competing needs

FGD participants were challenged to explain why so few IDPs owned a bed net, despite their free distribution. Extreme poverty among IDPs drives some to sell their bed nets, sometimes because of food insecurity. In the FGD involving health workers, one nurse explained: ‘*We ensure free bed net distribution to pregnant women and young children*, *but the issue is that many of these women sell their nets’ (Nurse)*. IDPs corroborated this explanation with their own, sometimes poignant, personal testimonials: *‘I used to use a bed net regularly before I became displaced*. *Nowadays*, *I don’t use it because I sold the net I received at the antenatal clinic because of food problems’ (IDP); ‘I traded [our bed net] for food because my children were hungry and I didn’t have money to solve this problem’ (IDP);‘We told our wives to sell the bed net they received in the market because we are heads of households without work*. *We can’t fulfill our basic duty*, *that’s why we sell some of our belongings so that the children can eat’ (IDP)*.

Further probing revealed that bed nets were not the only traded commodity. Participants reported that residents of nearby communities purchased a range of goods donated to the IDPs, and employed IDPs for inexpensive farming labour. Thus, one FGD participant from a neighboring community noted that bed nets could be purchased cheaply from IDPs: *‘At the market it costs around $2*.*5 but [from] IDPs the bed nets can be bought even for $0*.*5 [*…*] for me the cost is acceptable because even at the market it’s the price of one or two bottles of beer’ (Villager)*. Another noted the value-for-cost of donated items: *‘We buy several donated goods from the IDPs*, *because they sell them cheaper and the quality is good’ (Villager)*.Yet another noted the range of products available in the illicit market of donated goods:*‘[IDPs] sell goods at a cheap price*, *for example casseroles*, *tarps*, *and flour’ (Villager)*.In addition to demand for cheap quality goods, an appetite for a commerce in bed nets existed among IDPs on the “supply-side” as well: ‘*We sell our bed nets within the camp or at the market on Thursdays*, *because there are always lots of people in Thursday’ (IDP)*. Another participant described the widespread entrepreneurship around the bed net trade: ‘*Everyone buys bed nets*: *business people*, *NGO workers in the camp*, *even other IDPs in order to resell them when there is a shortage’ (IDP)*. The trade in bed nets was not condoned by authorities and, in one participant’s description, took on a humorously clandestine aspect: *‘Officially the sale [of bednets] is prohibited; that’s why the sale happens from ear to mouth or in a hidden corner of the market’* (IDP).

We further explored dynamics and tensions between IDPs and the neighboring community, which may influence the retention of bed nets in the camp. In addition to the benefit of low-price, high-quality donated goods, competing needs of IDPs and villagers sometimes lead to strain between the two populations. One FGD participant from a neighboring community noted: ‘*We get along easily*, *but these interactions also give rise to several conflicts*, *namely cases of rape*, *pregnancy*, *stealing of cattle or poultry’ (Villager)*. Another noted: *‘There is a big problem of theft in the village related to [IDP] presence’ (Villager)*. Selectively helping IDPs in a setting of widespread material poverty led to a perception of unfairness: *‘It’s unfair to ensure distribution of a necessary product only to displaced people knowing that we face the same nutritional difficulties as them (Villager)*. Another community member remarked on the burden borne by the neighboring village, and urged international donors not to forget surrounding communities in their zeal to help IDPs: *‘[We] encourage the donors in their goodwill [towards IDPs]*, *but [we ask that they] think of us as well because a house that has visitors spends [more] money’ (Villager)*.

The rich data generated from FGDs disclosed complexities in the use and disuse of malaria bed nets in and IDP camp. Of note, however, this very focus on bed nets was thrown into sharp relief by one participant’s sobering perspective on root causes of malaria: ‘*I think NGOs and the government should do everything in their power to return us to our villages by putting an end to the armed conflict and we will be able to fight against malaria because we will be in our own houses*, *[which are] better than a tent’ (IDP)*.

### Cross-sectional survey

#### Overall malaria control indicators

One hundred family units (“households”) in the IDP camp, consisting of 411 individuals, were surveyed between November, 2016 and March, 2017. Participants had been displaced for a median of 30 months (range 19–36 months) and had been living in the camp for most of this time (median 30 months, range 25–36 months). Indicators for malaria control in the IDP camp, based on standard survey indicators [25], are shown in Table 1. Overall, the burden of malaria was high (45/78 (58%) children <5 were positive for malaria by RDT), and bed net utilization was low (29/100 (29%) households owned a bed net, and 85/411 (20%) individuals slept under a bed net the previous night).

**Table 1:** Indicators for malaria control in an IDP camp^1^.

**Vector Control**	
Proportion of households with at least one bed net	29/100 (29%)
Proportion of households with at least one bed net for every two people (household access)[26]	0/100 (0%)
Proportion of population with access to a bed net within their household (population access)[26]	13%
Proportion of population that slept under a bed net the previous night	85/411 (20%)
Ratio of use to access	160%^2^
Proportion of children under five years old who slept under a bed net the previous night	32/78 (41%)
Proportion of existing bed nets used the previous night	29/29 (100%)
**Case management—Health seeking behaviour and accurate diagnosis**	
Proportion of children under five years old with fever in the last month for whom advice or treatment was sought	10/15 (67%)
Proportion of children under five years old with fever in the last month who had a RDT	10/15 (67%)
**Morbidity indicator:**	
Parasite Prevalence: proportion of children aged 6–59 months with *P*. *falciparum* infection	45/78 (58%)

^1^Modified from [25]

^2^Use:access ratio greater than 100% indicates that the mean number of users per net exceeded 2.0.[26]

#### Household composition, structure of shelter, and bed net ownership

Median household size was 4 individuals (range 2–8 individuals), with median 1 (range 1–2) child under 5. There was a median of 2 (range 1–4) shelters for each household. A positive correlation between the number of individuals per household and the number of shelters was observed (Spearman’s rho = 0.75, p<0.0001). Nearly all shelters were made of tarpaulin walls (94%), tarpaulin roof (100%), and mud/dirt floor (99%). No household had access to electricity, and all shared a latrine with other families. Households within the camp were characterized by extreme material poverty, with no households owning a refrigerator, television, car, or cow. Exposure to community violence was common, with at least one family member being victim of theft or physical violence in 48/100 (48%) households.

Twenty-nine (29%) households owned a bed net, and no household owned more than one bed net. No household had sufficient bed nets to accommodate all family members, allowing one bed net for every 2 individuals [25]. Each bed net was used by a median of 3 individuals (range 2 to 4), with no evidence of a behavioral gap in the use of bed nets among those who had access in the household [26]. Table 2 shows the characteristics of households, disaggregated by bed net ownership. Households with one or more children under 5 years of age were more likely to own a bed net than those without (27/69 (39%) vs 2/31 (6.5%), p = 0.0007). Households with a shelter made of durable material (mud walls) were more likely to own a bed net than those with tarpaulin walls (5/6 (87%) vs 24/94 (25%), p = 0.0075).

**Table 2:** Factors associated with household bed net ownership.

	Overall (N = 100)	Households with a bed net (N = 29)	Households without a bed net (N = 71)	p-value
**Duration of displacement in months, median (range)**	30 (19–36)	30 (29–36)	29 (19–35)	0.005
**Number of individuals per household, median (range)**	4 (2–8)	5 (3–8)	4 (2–7)	0.0019
**Number of children under 5 per household**				0.0001
None	31 (31%)	2 (6.9%)	29 (41%)	
One	60 (60%)	21 (72%)	39 (55%)	
Two	9 (9%)	6 (21%)	3 (4.2%)	
**Characteristics of shelter**				
**Flooring**				0.29
Dirt	99 (99%)	28 (97%)	71 (100%)	
Wood boards	1 (1%)	1 (3%)	0 (0%)	
**Roofing**				
Tarpaulin	100 (100%)	29 (100%)	71 (100%)	-
**Walls**				0.0075
Tarpaulin	94 (94%)	24 (83%)	70 (99%)	
Mud	6 (6%)	5 (17%)\	1 (1.5%)	
**Electricity**	0 (0%)	0 (0%)	0 (0%)	-
**Asset ownership**				
Refrigerator	0 (0%)	0 (0%)	0 (0%)	-
Television	0 (0%)	0 (0%)	0 (0%)	-
Radio	12 (12%)	3 (10%)	9 (13%)	>0.99
Cell phone^1^	2 (2%)	0 (0%)	2 (3%)	>0/99
Bicycle	8 (8%)	2 (7%)	6 (8%)	>0.99
Vehicle	0 (0%)	0 (0%)	0 (0%)	-
Watch^1^	13 (13%)	4 (14%)	9 (13%)	>0.99
Livestock	14 (14%)	2 (7%)	12 (17%)	0.34
**Wealth index**^2^				0.84
Above median	78 (78%)	23 (79%)	55 (77%)	
Below median	22 (22%)	6 (21%)	16 (23%)	

Values are n (%) unless stated otherwise.

^1^If any member of the household owned a cell phone or a watch, the household was classified as owning this asset.

^2^Assets that did not contribute to the wealth index include those that were not owned by any household, namely refrigerator, television, and vehicle.

#### Survey participants, *P*. *falciparum* infection, and bed net use

The median age of the 411 study participants was 23 (range 1–75), 78/411 (19%) were under the age of 5, and 177/411 (43%) were female. At the time of the survey, 61 (15%), 50 (12%), and 26 (6%) of individuals were symptomatic with headache, fever, and/or myalgia, respectively, and 69 (17%) tested positive for malaria by RDT. Symptoms were statistically significantly associated with RDT positivity (p <0.0001 for all comparisons). In the target group of children under 5, fever was reported in 40/78 (51%) and RDT was positive in 45/78 (58%) at the time of the survey. Thus, age<5 was associated with higher odds of fever (OR 16 (95%CI 8.4–29) p<0.0001) and RDT positivity (OR 18 (95%CI 9.5–32), p<0.0001), relative to older children and adults. Because RDT positivity may persist for up to 4 weeks after resolved *P*. *falciparum* infection [27,28], we performed a subgroup analysis to account for possible false-positive results. RDT was positive in 6/15 (40%) of children with a reported fever in the past month, and 39/63 (62%) of children without a reported fever in the past month (p = 0.15).

The night prior to the survey, 85/411 (20%) individuals had slept under a bed net, including 31/78 (40%) children under 5. Children under 5 were more likely to use a bed net than older children or adults (OR 3.4 (95%CI 2.0–5.8), p<0.0001). Table 3 shows individual-level characteristics, disaggregated by bed net use. Besides age, no other factors were associated with bed net use.

**Table 3:** Individual-level factors associated with bed net use.

	Overall	Used bed net^1^	Did not use bed net	p-value
***All participants (N = 411)***		**N = 85**	**N = 326**	
**Age, median (range)**	23 (1–75)	16 (1–36)	24 (1–75)	0.002
**Sex**				0.69
Male	234 (57%)	50 (59%)	184 (56%)	
Female	177 (43%)	35 (41%)	142 (44%)	
***Children under 5 (N = 78)***		**N = 31**	**N = 47**	
**Age, median (range)**	2.5 (1–4)	2.6 (1–4)	2.4 (1–4)	0.24
**Sex**				0.44
Male	54 (69%)	23 (74%)	31 (66%)	
Female	24 (31%)	8 (26%)	16 (34%)	
**Maternal education**				0.85
None	39 (50%)	16 (51%)	23 (49%)	
Primary	17 (22%)	8 (26%)	9 (19%)	
Secondary and above	22 (28%)	7 (23%)	15 (32%)	
**Symptoms**^2^				
Fever	40 (51%)	17 (55%)	23 (49%)	0.61
Headache	8 (10%)	5 (16%)	3 (6%)	0.25
Myalgia	9 (12%)	4 (13%)	5 (11%)	>0.99
**RDT positive result**	45 (58%)	18 (58%)	27 (57%)	0.96
**Fever in the past month**	15 (19%)	5 (16%)	10 (21%)	0.77
Sought care	10 (67%)	4 (80%)	6 (60%)	0.15
Diagnosed with malaria	10 (67%)	4 (80%)	6 (60%)	0.15
Tested with RDT or microscopy	10 (67%)	4 (80%)	6 (60%)	0.15
Received antimalarial	10 (67%)	4 (80%)	6 (60%)	0.15

Values are n (%) unless stated otherwise.

^1^Bed net use is defined as sleeping under the bed net the night prior to the questionnaire.

^2^Symptoms reported by participants at the time of the questionnaire were recorded.

#### Bed nets currently in use

Of the 29 bed nets currently in use by study participants, 21 (72%) were obtained free of charge at the IDP camp health center during an ANC visit, and 8 (28%) were received free of charge from NGOs. No bed nets had been purchased. The median duration since the net was received was 25 months (range 14–30 months). Most (21/29 (72%)) of the nets were washed regularly and 15/29 (52%) had developed holes.

#### Bed nets no longer used

Asked to recall any bed nets they had owned since coming to the IDP camp, participants reported a total of 146 bed nets that they no longer owned. Of these, 120 (82%) were received from NGOs and 26 (18%) were received from the IDP camp health center. None of the bed nets had been purchased. All of these nets had been sold (82%) or exchanged (18%) either in the camp (27%) or in the neighboring village market (73%) at a median price of $2.15 (range $1–3). None had been discarded, repurposed, or worn out. The majority of nets were bought or accepted by residents of the neighboring community (80%), however other IDP camp residents (12%) and government workers (8%) also accepted or bought the nets. The numerous reasons given for selling or exchanging a bed net are given in Table 4. The most common reasons related to pragmatic considerations including installation and size of the net, rather than lack of awareness of their utility, or monetary value of the net.

**Table 4:** Factors associated with bed net disuse.

	Bed nets previously owned^1^ (N = 146)
**Reasons for bed net disuse**	
Causes irritations/coughing	100 (68%)
Not shaped well	78 (53%)
Difficult installation	74 (51%)
Gives off a chemical	70 (48%)
Too many holes	64 (44%)
Smells badly	54 (37%)
Gets dirty quickly	43 (29%)
Needed money	24 (16%)
Too small	23 (16%)
Too hot	16 (11%)
Can suffocate/causes difficulties breathing	0 (0%)
Can’t tuck under mattress	0 (0%)
Causes illness	0 (0%)
Causes nausea	0 (0%)
Not efficacious	0 (0%)
Other	0 (0%)
Don’t know	0 (0%)
**Outcome of net**	
Sold	119 (82%)
Exchanged	27 (18%)
**Duration since exchange/selling of net**	26 (2–34)
**Place of exchange/selling of net**	
Village market	107 (73%)
Camp	39 (27%)
**Who bought/accepted net**	
Villager	117 (80%)
Displaced individual	17 (12%)
Government worker	12 (8%)
NGO	0 (0%)
**Price net was sold for, median (range)**	$2.15 (1–3)

Values are n (%) unless stated otherwise.

^1^Previous bed nets are those that households had reported owning since their displacement but had now been sold or exchanged.

Table 5 compares bed nets owned and in use at the time of the survey to those that had been sold or exchanged. Bed nets received at the health centre were more likely to still be in use than those received from an NGO (OR 12.1, 95%CI 10.7–18.9). Bed nets received during an antenatal care visit were more likely to still be owned and in use (OR 13.3, 95%CI 5.3–33.6).

**Table 5:** Factors associated with use of bed nets.

	Bed nets currently owned^1^ (N = 29)	Bed nets previously owned^2^ (N = 146)	p-value
**Age of bed net in months, median (range)**	25 (14–30)	28 (12–39)	0.012
**Source of bed net**			<0.0001
NGO	8 (28%)	120 (82%)	
Hospital/ Health Centre	21 (72%)	26 (18%)	
**Received during ANC visit**	21 (72%)	24 (16%)	<0.0001
**Received during distribution campaigns**	-	119 (82%)	-

Values are n (%) unless stated otherwise.

^1^Current bed nets are those that were used the night prior to the questionnaire.

^2^Previous bed nets are those that households had reported owning since their displacement to the IDP camp but had now been sold or exchanged.

## Discussion

Our study of bed net use and disuse in an IDP camp in Eastern DRC unveils complexities and limitations of an established malaria control strategy applied in a challenging environment. Studies from this area and among displaced populations are scarce; therefore, these data offer rare insights into an under-studied population. Our findings may be useful to governments and NGOs operating in complex humanitarian crises in the tropics, where malaria is exacerbated by violence and displacement [9], and plays a major role in child mortality [11,15,29–32].

An important element of malaria prevention is the use of vector control measures, including bed nets. These act as a physical and chemical barrier to night-biting female *Anopheles* mosquitoes and are highly effective in field trials [33,34]; however, it is less clear how to deploy this strategy in the challenging environment of an IDP camp. In this study, we investigated barriers to bed net use among IDPs in a region with ongoing human insecurity. Despite a high burden of malaria and free distribution of bed nets, only 29% of IDP households owned a bed net, compared to 16–75% in other studies in IDP camps in Eastern DRC and 8–90% in community-based surveys and surveillance data from sub-Saharan Africa [9,11,13,14,35]. In our survey of an IDP camp in Eastern DRC, 20% of individuals slept under a bed net the previous night, compared to 16–25% in another IDP camp [9] and 6–70% in other African studies [36]. Point prevalence of *P*. *falciparum* infection among children under 5 in our study was 58%, compared to 0.4–78% in other studies in Sub-Saharan Africa [11,36]. Taken together, these data indicate that the Birambizo IDP camp appears to be representative of other IDP camps in the area, with high malaria burden and low bed net use.

A large number (146/175 (83%)) of the total number of bed nets reported by IDP camp residents had been sold or exchanged and were no longer available for malaria prevention in the population for which they were intended. The reasons given for dispensing of a bed net were solicited in both FGDs (qualitative methodology) and survey questionnaires (quantitative methodology). The reasons given were protean (Table 5). Lack of awareness of malaria burden or bed net effectiveness did not emerge in either FGDs or surveys as a major reason for dispensing of bed nets. Unlike participants from a community-based survey in Kenya [37], no participants in our study reported repurposing their bed net for other uses such as a blanket or curtain.

Both qualitative and quantitative methods pointed to substantial pragmatic limitations of bed nets in the IDP camp. Sleeping arrangements (multiple shelters, lack of beds/mattresses), cramped space inside the tent, mud floors and need for frequent washing, difficulties in hanging a bed net inside, and the development of holes were given as practical reasons why bed nets may not be used. Similar pragmatic barriers have been previously identified in community-based African studies exploring bed net underutilization [38,39]. Characteristics of the bed nets themselves, independent of the IDP camp context (e.g., unpleasant odor or chemical irritation) were other common reasons for bed net disuse. These reasons are consistent with other community survey results in African countries with free insecticide-treated bed nets (ITN) and long-lasting insecticide treated net (LLIN) distribution campaigns [38–40]. Due to a lack of space within the camp and an immediate need for the nets, new bed nets are likely to be used without following manufacturer’s instructions for a 24–48 hour drying period, which may be responsible for the initial irritations and chemical odor reported by FGD and survey participants.

In FGDs, the position of economic vulnerability of IDPs, relative to neighboring communities, appeared to fuel a trade in bed nets and other donated goods. Selling bed nets to fill an immediate need for food or income was a noteworthy finding; however, quantitative survey results suggest that this was the reason for dispensing of only 16% of the bed nets that were no longer in use. We suspect that social acceptability bias may have led to an under-reporting of financial motives for bed net sale/trade in survey questionnaires. Of note, selling or trading nets is strongly discouraged by authorities, and door-to-door surveys were conducted by members of the health center. On the other hand, FGDs were led by IDP camp members themselves with special attention to participant engagement and confidentiality; therefore participants may have been more open and honest in their discussions.

Commercial transactions in donated goods within the IDP camp and with the surrounding community explained, at least in part, the large number of bed nets that had been sold or exchanged. FGDs revealed a dynamic exchange of goods and services between the camp and neighboring community, including farm labour, exchange of donated quality household goods, including bed nets. Most bed nets were sold to neighboring villagers, at a price well below retail value. In addition to sympathy for the general plight of the IDPs, resentments were expressed by some FGD participants from the surrounding community, who were not beneficiaries of free commodities distributed in the camp by external agents (NGOs), and who sometimes experienced theft at the hands of the IDPs. Recognizing that freely distributed bed nets were not supposed to be sold or bartered, the trade in donated bed nets was often clandestine, from “mouth to ear” or in a “hidden corner of the market.”

Children under 5 carry a disproportionate burden of malaria [30,31,41]; this was reflected in our study where fever and RDT positivity were more prevalent in this age group. Appropriately, bed nets were preferentially used for this age group, as evidenced by higher household ownership of bed nets in households with at least one child under 5, and higher utilization of bed nets by children under 5. Bed net distribution during ANC visits, as done in the IDP camp Health Centre and in other IDP camps and regions of Africa [42–44], appears to be a rational strategy to target the youngest camp residents, and appears to be at least partially successful in the Birambizo IDP camp. Higher rates of bed net retention were observed when bed nets were obtained in the context of an antenatal clinic visit. Still, there remain significant gaps in bed net coverage for the under 5 age group, since 47/78 (60%) children under 5 did not sleep under a bed net the night prior to the survey and the burden of malaria was concentrated in this group.

As highlighted by both the qualitative and quantitative results presented here, barriers to bed net use among IDPs are numerous, and call into question the usefulness of bed net distribution campaigns as currently implemented in IDP camps in sub-Saharan Africa. Bed nets in more stable contexts in Africa are an evidence-based approach to malaria control with proven effectiveness, ease of use and distribution [1,34]. However, in the context of complex humanitarian emergencies where households are mobile, living conditions are cramped, and malaria transmission is elevated, bed net distribution campaigns may be less effective. Modifications to bed net design may make them more user-friendly within the small shelters of displaced populations. Alternatively, indoor residual spraying (IRS) could be used; however IRS can be operationally challenging and economically unsustainable [1,45–47]. Within Birambizo IDP camp, IRS was carried out when NGOs were permanently operating within the camp, however violent conflict has interrupted NGO efforts at maintaining IRS interventions. One other possible vector control method that could be well suited to the IDP camp setting is the use of insecticide-treated tarpaulin for IDP shelters. A recent review [45] highlights evidence suggesting the effectiveness of insecticide-treated durable wall lining over multiple years, however phase III trials are still underway to provide evidence as to the operational implementation of this novel vector control method within displaced populations [48–50].

Our study is noteworthy as a qualitative description of barriers to bed net utilization and a quantitative description of RDT-confirmed malaria in an IDP camp deep within the zone of ongoing human insecurity. Nonetheless, this study has several limitations. It was conducted at a single, densely populated IDP camp within a region of high malaria transmission. Therefore, results should not be extrapolated to low transmission settings or areas with marked seasonal malaria transmission. The wealth index is specific for the IDP context and cannot be compared to other wealth index due to the extreme poverty and subsequently low asset ownership of IDPs. Furthermore, it is well-recognized that the HRP2-based RDT (Paracheck-Pf^®^) used in our cross-sectional study will test positively up to four weeks after parasite clearance [27,28], such that individuals who had infections in the past month would test positively. Additionally, the HRP2-based RDT may have limited sensitivity at low parasite density, which would lead to underestimations in point prevalence particularly among non-symptomatic participants [51]. The use of additional diagnostic tools such as microscopy and/or polymerase chain reaction would be desirable; however, given the resource limitations in our study setting, this was not feasible. Lastly, emerging insecticide resistance of mosquitoes [1] was not addressed, but could be a factor threatening the efficacy of bed nets within the context of our study population, where bed nets are not consistently used and bed net distribution campaigns are unpredictable.

## Conclusions

This study, from a complex chronic humanitarian crisis in the DRC, illustrates complexities and limitations of bed net distribution as a malaria control strategy in an IDP camp. Engineering improvements on current bed net design, to accommodate the special needs of a tented camp, might be considered to improve their acceptability, ease of use, and retention in this unique setting. Alternative vector control methods (e.g., larval breeding control, indoor residual spraying, insecticide-treated tarpaulin, and others) may be considered in addition to or in lieu of bed nets in the IDP camp setting. Addressing competing needs and food insecurity will be important to ensure that donated goods, including bed nets, are not sold or exchanged.

IDPs in remote areas in the tropics, further isolated by violent conflict, and afflicted by malaria among numerous other threats to their health and well-being, pose a formidable public health challenge. Given the unique situation in an IDP camp, malaria control strategies borrowed from other contexts may not be directly transferrable. Our findings call for an improved, thoughtful, and tailored approach to address the burden of malaria in IDP camps in the tropics and suggest that bed net distribution alone is not a panacea.

## Supporting information

S1 Dataset Cross-sectional survey dataset. A random sample of 100 households in Birambizo IDP camp were surveyed, answers were recorded and coded into excel. Each worksheet in the dataset represents a different table in the questionnaire. Names of participants were removed for ethical reasons.
(XLSX)

S1 Table Focus group discussion participants and criteria. Participants were purposively selected by chiefs of the IDP camp and villages, to match our criteria.
(PDF)

S2 Table Malaria indicator survey (French). Community health workers visited a random sample of 100 households in Birambizo IDP camp, asking these questionnaire questions and recording participant answers. The questionnaire was originally created in French, as seen here.
(PDF)

S3 Table Malaria indicator survey (English). Community health workers visited a random sample of 100 households in Birambizo IDP camp, asking these questionnaire questions and recording participant answers. The questionnaire was originally created in French and has been translated to English.
(PDF)
