# Supplementary material for: Use and disuse of malaria bed nets in an internally displaced persons camp in the Democratic Republic of the Congo: A mixed-methods study
Source: PLoS One. 2017 Sep 26;12(9):e0185290. doi: 10.1371/journal.pone.0185290 (PMC5614551; doi:10.1371/journal.pone.0185290)
Supplement: S1 Table — Participants were purposively selected by chiefs of the IDP camp and villages, to match our criteria. (PDF) [file pone.0185290.s002.pdf]

S1 Table. Focus group discussion participants and criteria.

	Number of Individuals (N=55)	Residence	Sex	Children < 5 in the residence	Bednet Ownership
**FGD 1**	6	IDP camp	F	Yes	Yes
**FGD 2**	6	IDP camp	F	Yes	No
**FGD 3**	6	IDP camp	M	Yes	No
**FGD 4**	3	IDP camp	M	Yes	-
	3	IDP camp	F	Yes	-
**FGD 5**	3	IDP camp	F	Yes	Yes
	3	IDP camp	F	Yes	No
**FGD 6**	3	IDP camp	F	Yes	Yes
	3	Village	F	Yes	Yes
**FGD 7**	3	IDP camp	F	Yes	No
	3	Village	F	Yes	No
**FGD 8**	5	IDP camp	F	Yes	No
**FGD 9**	5	Village	F	-	-
**FGD 10**	3	Village (Nurse)	-	-	-
