# Supplementary material for: Use and disuse of malaria bed nets in an internally displaced persons camp in the Democratic Republic of the Congo: A mixed-methods study
Source: PLoS One. 2017 Sep 26;12(9):e0185290. doi: 10.1371/journal.pone.0185290 (PMC5614551; doi:10.1371/journal.pone.0185290)
Supplement: S2 Table — Community health workers visited a random sample of 100 households in Birambizo IDP camp, asking these questionnaire questions and recording participant answers. The questionnaire was originally created in French, as seen here. (PDF) [file pone.0185290.s003.pdf]

SONDAGE : INDICATEURS DU PALUDISME

DATE____ ENQUÊTEUR(TRICE)____

NOM DE LA LOCALITÉ____ No. ENREGISTREMENT DU MENAGE_____________________________

COMPOSITION DES MÉNAGES

LIGNE NO.	MEMBRES HABITUELS ET VISITEURS	RELATION AU CHEF DE MENAGE	SEXE	AGE	RESIDENCE		MOUSTIQUAIRE	PALUSIDME		VIOLENCE
1	2	3	4	5	6	7	8	9	10	11
	S'il vous plait, donnez-moi les noms des personnes qui vivent habituellement dans votre ménage et des visiteurs qui ont passé la nuit dernière ici, en commençant par le chef de ménage.<br>APRÈS AVOIR LISTÉ NOMS ET ENREGISTRÉ LE LIEN DE PARENTÉ ET LE SEXE POUR CHAQUE PERSONNE, POSEZ LES QUESTIONS 2A-2C POUR VOUS ASSURER QUE LA LISTE EST COMPLÈTE.<br>POSEZ ENSUITE LES QUESTIONS APPROPRIÉES DES COLONNES 5-11 POUR CHAQUE PERSONNE.	Quel est le lien de parenté de (NOM) avec le chef de ménage ?<br>VOIR CODES CI-DESSOUS	(NOM) est-il de sexe masculin ou féminin?	Quel âge a (NOM) ?<br>NOTER EN ANNÉES	Est-ce que (NOM) dors normalement dans cette tente/abris ?	Est-ce que (NOM) a dormi dans cette tente/abris hier soir?	Est-ce que (NOM) a dormi sous une moustiquaire hier soir?	Est-ce que (NOM) a un ou plusieurs des symptôme suivant aujourd'hui ?	Résultat du test rapide ?	Est-ce que (NOM) a été victime d'un ou plusieurs des suivant, depuis être déplacée ?
01			M ☐<br>F ☐		OUI ☐<br>NON ☐	OUI ☐<br>NON ☐		CÉPHALÉE ☐<br>MYALGIE ☐<br>FIÈVRE ☐	TDR + ☐<br>TDR - ☐	VOL ☐<br>AGRESSION ☐<br>AGRESSION SEXUEL/VIOL ☐<br>POIGNARDEZ ☐<br>FUSILLEZ ☐
02			M ☐<br>F ☐		OUI ☐<br>NON ☐	OUI ☐<br>NON ☐		CÉPHALÉE ☐<br>MYALGIE ☐<br>FIÈVRE ☐	TDR + ☐<br>TDR - ☐	VOL ☐<br>AGRESSION ☐<br>AGRESSION SEXUEL/VIOL ☐<br>POIGNARDEZ ☐<br>FUSILLEZ ☐
03			M ☐<br>F ☐		OUI ☐<br>NON ☐	OUI ☐<br>NON ☐		CÉPHALÉE ☐<br>MYALGIE ☐<br>FIÈVRE ☐	TDR + ☐<br>TDR - ☐	VOL ☐<br>AGRESSION ☐<br>AGRESSION SEXUEL/VIOL ☐<br>POIGNARDEZ ☐<br>FUSILLEZ ☐

04			M ☐<br>F ☐		OUI ☐<br>NON ☐	OUI ☐<br>NON ☐		CÉPHALÉE ☐<br>MYALGIE ☐<br>FIÈVRE ☐	TDR +☐<br>TDR - ☐	VOL ☐<br>AGRESSION ☐<br>AGRESSION SEXUEL/VIOL ☐<br>POIGNARDEZ ☐<br>FUSILLEZ ☐
05			M ☐<br>F ☐		OUI ☐<br>NON ☐	OUI ☐<br>NON ☐		CÉPHALÉE ☐<br>MYALGIE ☐<br>FIÈVRE ☐	TDR +☐<br>TDR - ☐	VOL ☐<br>AGRESSION ☐<br>AGRESSION SEXUEL/VIOL ☐<br>POIGNARDEZ ☐<br>FUSILLEZ ☐
06			M ☐<br>F ☐		OUI ☐<br>NON ☐	OUI ☐<br>NON ☐		CÉPHALÉE ☐<br>MYALGIE ☐<br>FIÈVRE ☐	TDR +☐<br>TDR - ☐	VOL ☐<br>AGRESSION ☐<br>AGRESSION SEXUEL/VIOL ☐<br>POIGNARDEZ ☐<br>FUSILLEZ ☐
07			M ☐<br>F ☐		OUI ☐<br>NON ☐	OUI ☐<br>NON ☐		CÉPHALÉE ☐<br>MYALGIE ☐<br>FIÈVRE ☐	TDR +☐<br>TDR - ☐	VOL ☐<br>AGRESSION ☐<br>AGRESSION SEXUEL/VIOL ☐<br>POIGNARDEZ ☐<br>FUSILLEZ ☐
08			M ☐<br>F ☐		OUI ☐<br>NON ☐	OUI ☐<br>NON ☐		CÉPHALÉE ☐<br>MYALGIE ☐<br>FIÈVRE ☐	TDR +☐<br>TDR - ☐	VOL ☐<br>AGRESSION ☐<br>AGRESSION SEXUEL/VIOL ☐<br>POIGNARDEZ ☐<br>FUSILLEZ ☐
09			M ☐<br>F ☐		OUI ☐<br>NON ☐	OUI ☐<br>NON ☐		CÉPHALÉE ☐<br>MYALGIE ☐<br>FIÈVRE ☐	TDR +☐<br>TDR - ☐	VOL ☐<br>AGRESSION ☐<br>AGRESSION SEXUEL/VIOL ☐<br>POIGNARDEZ ☐<br>FUSILLEZ ☐
10			M ☐<br>F ☐		OUI ☐<br>NON ☐	OUI ☐<br>NON ☐		CÉPHALÉE ☐<br>MYALGIE ☐<br>FIÈVRE ☐	TDR +☐<br>TDR - ☐	VOL ☐<br>AGRESSION ☐<br>AGRESSION SEXUEL/VIOL ☐<br>POIGNARDEZ ☐<br>FUSILLEZ ☐

2A) Juste pour être sûr que j'ai une liste complète : y-a-t-il d'autres personnes telles que des petits enfants ou des nourrissons que nous n'avons pas listées? (Si oui, ajouter au tableau)
2B) Y-a-t-il d'autres personnes qui ne sont peut-être pas membres de votre famille, tels que des domestiques, locataires ou amis qui vivent habituellement ici ? (Si oui, ajouter au tableau)
2C) Avez-vous des invités ou des visiteurs temporaires qui sont chez vous, ou d'autres personnes qui ont dormi ici la nuit dernière et qui n'ont pas été listées? (Si oui, ajouter au tableau)

CODES POUR Q. 3: LIEN AVEC LE CHEF DU MÉNAGE					
	01 = CHEF DE MÉNAGE	04 = GENDRE/BELLE-FILLE	07 = BEAUX-PARENTS	10 = ADOPTÉ/EN GARDE	12 = CO-EPOUSE
	02 = FEMME OU MARI	05= PETIT-FILS/FILLE	08 = FRÈRE OU SOEUR	11 = ENFANTS DE LA FEMME/MARI	13 = SANS PARENTÉ
	03 =FILS OU FILLE	06 = PÈRE/MÈRE	09 = AUTRE PARENT		98 = NE SAIT PAS

ENFANTS DE MOINS DE 5 ANS

LIGNE NO.	NOMS DES ENFANTS MOINS DE 5 ANS	FIEVRE		CONSEILS MEDICALE		PALUDISME		
1	2	3	4	5	6	7	8	9
11	S'il vous plait donner les noms des enfants moins de 5 ans et le ligne numéro à partir du tableau de la composition des ménages.	Est-ce que l'enfant a eu une fièvre dans le mois précédent?	Si oui, avez-vous recherché des conseils ou traitement au centre médical ?	Est-ce que l'enfant avait le paludisme ?	Si oui, cas de palu confirmé par test rapide ou microscope?	Est-ce que le traitement antipaludique été donné?	Quel est le niveau d'instruction de la mère?	La mère, est-elle alphabétisée?
12	NOM_______________<br>LIGNE NO. ☐	OUI ☐<br>NON☐	OUI ☐<br>NON☐	OUI ☐<br>NON☐	OUI ☐<br>NON☐	OUI ☐<br>NON☐	AUCUN ☐<br>PRIMAIRE ☐<br>SECONDAIRE☐<br>SUPÉRIEUR ☐	OUI ☐<br>NON☐
13	NOM_______________<br>LIGNE NO. ☐	OUI ☐<br>NON☐	OUI ☐<br>NON☐	OUI ☐<br>NON☐	OUI ☐<br>NON☐	OUI ☐<br>NON☐	AUCUN ☐<br>PRIMAIRE ☐<br>SECONDAIRE☐<br>SUPÉRIEUR ☐	OUI ☐<br>NON☐
14	NOM_______________<br>LIGNE NO. ☐	OUI ☐<br>NON☐	OUI ☐<br>NON☐	OUI ☐<br>NON☐	OUI ☐<br>NON☐	OUI ☐<br>NON☐	AUCUN ☐<br>PRIMAIRE ☐<br>SECONDAIRE☐<br>SUPÉRIEUR ☐	OUI ☐<br>NON☐
15	NOM_______________<br>LIGNE NO. ☐	OUI ☐<br>NON☐	OUI ☐<br>NON☐	OUI ☐<br>NON☐	OUI ☐<br>NON☐	OUI ☐<br>NON☐	AUCUN ☐<br>PRIMAIRE ☐<br>SECONDAIRE☐<br>SUPÉRIEUR ☐	OUI ☐<br>NON☐
16	NOM_______________<br>LIGNE NO. ☐	OUI ☐<br>NON☐	OUI ☐<br>NON☐	OUI ☐<br>NON☐	OUI ☐<br>NON☐	OUI ☐<br>NON☐	AUCUN ☐<br>PRIMAIRE ☐<br>SECONDAIRE☐<br>SUPÉRIEUR ☐	OUI ☐<br>NON☐

MOUSTIQUAIRES

LIGNE NO.		MOUSTIQUAIRE #1	MOUSTIQUAIRE #2	MOUSTIQUAIRE #3
17	DEMANDE A VOIR TOUS LES MOUSTIQUAIRES DANS LA MÉNAGE<br><br>SI PLUS DE 3 MOUSTIQUAIRES, UTILISEZ UN/DES QUESTIONNAIRES SUPPLÉMENTAIRES	OBSERVÉE ☐<br>PAS OBSERVÉE ☐	OBSERVÉE ☐<br>PAS OBSERVÉE ☐	OBSERVÉE ☐<br>PAS OBSERVÉE ☐
18	CA FAIT COMBIEN DE MOIS QUE VOTRE MÉNAGE A LA MOUSTIQUAIRE?	EN MOIS ☐	EN MOIS ☐	EN MOIS ☐
19	EST-CE QUE VOUS AVEZ REÇU LA MOUSTIQUAIRE PAR UNE CAMPAGNE DE DISTRIBUTION QUAND VOUS ETTE ARRIVÉE AU CAMP	OUI ☐<br>NON ☐<br>NE SAIT PAS ☐	OUI ☐<br>NON ☐<br>NE SAIT PAS ☐	OUI ☐<br>NON ☐<br>NE SAIT PAS ☐
20	EST-CE QUE VOUS AVEZ REÇU LA MOUSTIQUAIRE OU PENDANT UNE VISITE PRÉNATALS?	OUI ☐<br>NON ☐<br>NE SAIT PAS ☐	OUI ☐<br>NON ☐<br>NE SAIT PAS ☐	OUI ☐<br>NON ☐<br>NE SAIT PAS ☐
21	OU AVEZ-VOUS OBTENU LA MOUSTIQUAIRE?	HOPITAL/CENTRE DE SANTÉ/POSTE DE SANTÉ PUBLIC ☐<br><br>HOPITAL/CLINIQUE PRIVE ☐<br><br>PHARMACIE ☐<br><br>MARCHÉ ☐<br><br>AGENT DE SANTÉ COMMUNAUTAIRE ☐<br><br>INSTITUT RELIGIEUX ☐<br><br>ÉCOLE ☐<br><br>AUTRE (précisez) ☐<br>________________<br><br>NE SAIT PAS ☐	HOPITAL/CENTRE DE SANTÉ/POSTE DE SANTÉ PUBLIC ☐<br><br>HOPITAL/CLINIQUE PRIVE ☐<br><br>PHARMACIE ☐<br><br>MARCHÉ ☐<br><br>AGENT DE SANTÉ COMMUNAUTAIRE ☐<br><br>INSTITUT RELIGIEUX ☐<br><br>ÉCOLE ☐<br><br>AUTRE (précisez) ☐<br>________________<br><br>NE SAIT PAS ☐	HOPITAL/CENTRE DE SANTÉ/POSTE DE SANTÉ PUBLIC ☐<br><br>HOPITAL/CLINIQUE PRIVE ☐<br><br>PHARMACIE ☐<br><br>MARCHÉ ☐<br><br>AGENT DE SANTÉ COMMUNAUTAIRE ☐<br><br>INSTITUT RELIGIEUX ☐<br><br>ÉCOLE ☐<br><br>AUTRE (précisez) ☐<br>________________<br><br>NE SAIT PAS ☐
22	EST-CE QUE VOUS UTILISEZ LA MOUSTIQUAIRE?<br><br>SI OUI, ALLEZ A Q 24, SI NON DEMANDE Q 23 ET ALLEZ A Q 31.	OUI ☐<br>NON ☐<br>NE SAIT PAS ☐	OUI ☐<br>NON ☐<br>NE SAIT PAS ☐	OUI ☐<br>NON ☐<br>NE SAIT PAS ☐

23	POURQUOI VOTRE MÉNAGE N'UTILISE-T-IL PAS LA MOUSTIQUAIRE ?<br><br>ENREGISTREZ TOUT CE QUI EST MENTIONNE<br><br>INSISTEZ : AUTRES RAISON ?	MOUSTIQUAIRE PAS EFFICACE ☐<br>CERTAINS MEMBRES DU MENAGE N'AIMENT PAS LES MOUSTIQUAIRES ☐<br>TAILLE NON SATISFAISANTE ☐<br>FORME NON SATISFAISANTE ☐<br>MAUVAISE ODEUR ☐<br>CAUSE DES IRRITATIONS/ TOUX ☐<br>REND MALADE ☐<br>DONNE DES NAUSÉES ☐<br>PRODUIT CHIMIQUE DANGEREUX ☐<br>PEUT SUFFOQUER/DIFFICULTÉS RESPIRER ☐<br>CHALEUR ☐<br>MOUSTIQUAIRE SE SALIT VITE ☐<br>PAS DE RAISON ☐<br>AUTRE (précisez)☐<br>__________________<br>NE SAIT PAS ☐	MOUSTIQUAIRE PAS EFFICACE ☐<br>CERTAINS MEMBRES DU MENAGE N'AIMENT PAS LES MOUSTIQUAIRES ☐<br>TAILLE NON SATISFAISANTE ☐<br>FORME NON SATISFAISANTE ☐<br>MAUVAISE ODEUR ☐<br>CAUSE DES IRRITATIONS/ TOUX ☐<br>REND MALADE ☐<br>DONNE DES NAUSÉES ☐<br>PRODUIT CHIMIQUE DANGEREUX ☐<br>PEUT SUFFOQUER/DIFFICULTÉS RESPIRER ☐<br>CHALEUR ☐<br>MOUSTIQUAIRE SE SALIT VITE ☐<br>PAS DE RAISON ☐<br>AUTRE (précisez)☐<br>__________________<br>NE SAIT PAS ☐	MOUSTIQUAIRE PAS EFFICACE ☐<br>CERTAINS MEMBRES DU MENAGE N'AIMENT PAS LES MOUSTIQUAIRES ☐<br>TAILLE NON SATISFAISANTE ☐<br>FORME NON SATISFAISANTE ☐<br>MAUVAISE ODEUR ☐<br>CAUSE DES IRRITATIONS/ TOUX ☐<br>REND MALADE ☐<br>DONNE DES NAUSÉES ☐<br>PRODUIT CHIMIQUE DANGEREUX ☐<br>PEUT SUFFOQUER/DIFFICULTÉS RESPIRER ☐<br>CHALEUR ☐<br>MOUSTIQUAIRE SE SALIT VITE ☐<br>PAS DE RAISON ☐<br>AUTRE (précisez)☐<br>__________________<br>NE SAIT PAS ☐
24	DEPUIS QUE VOUS AVEZ LA MOUSTIQUAIRE, A-T-IL DÉVELOPPÉ DES TROUS?	OUI ☐<br>NON ☐<br>NE SAIT PAS ☐	OUI ☐<br>NON ☐<br>NE SAIT PAS ☐	OUI ☐<br>NON ☐<br>NE SAIT PAS ☐
25	SI OUI, COMBIEN DE TROUS A-T-IL?	BEAUCOUP ☐<br>CERTAINS ☐<br>QUELQUES-UN ☐	BEAUCOUP ☐<br>CERTAINS ☐<br>QUELQUES-UN ☐	BEAUCOUP ☐<br>CERTAINS ☐<br>QUELQUES-UN ☐
26	EST-CE QUE VOUS LAVEZ LA MOUSTIQUAIRE?	OUI ☐<br>NON ☐<br>NE SAIT PAS ☐	OUI ☐<br>NON ☐<br>NE SAIT PAS ☐	OUI ☐<br>NON ☐<br>NE SAIT PAS ☐
27	SI OUI, CA FAIT COMBIEN DE JOURS DEPUIS LE DERNIER LAVAGE DE LA MOUSTIQUAIRE?	EN JOURS ☐	EN JOURS ☐	EN JOURS ☐
28	DEPUIS QUE VOUS AVEZ OBTENU CETTE MOUSTIQUAIRE, ÉTAIT-ELLE DÉJÀ TRAITEE AVEC UN INSECTICIDE POUR TUER OU ELOIGNER LES MOUSTIQUES ?	OUI ☐<br>NON ☐<br>NE SAIT PAS ☐	OUI ☐<br>NON ☐<br>NE SAIT PAS ☐	OUI ☐<br>NON ☐<br>NE SAIT PAS ☐

29	LA NUIT DERNIERE, QUELQU'UN A DORMI SOUS CETTE MOUSTIQUAIRE?	OUI ☐<br>NON ☐<br>NE SAIT PAS ☐	OUI ☐<br>NON ☐<br>NE SAIT PAS ☐	OUI ☐<br>NON ☐<br>NE SAIT PAS ☐
30	SI OUI, QUI A DORMI SOUS CETTE MOUSTIQUAIRE HIER SOIR ?<br><br>ENREGISTREZ LE NOM ET LIGNE NUMERO A PARTIR DU TABLEAU DE LA COMPOSITION DES MÉNAGES.	NOM_______________<br>LIGNE NO. ☐<br><br>NOM_______________<br>LIGNE NO. ☐<br><br>NOM_______________<br>LIGNE NO. ☐<br><br>NOM_______________<br>LIGNE NO. ☐<br><br>NOM_______________<br>LIGNE NO. ☐	NOM_______________<br>LIGNE NO. ☐<br><br>NOM_______________<br>LIGNE NO. ☐<br><br>NOM_______________<br>LIGNE NO. ☐<br><br>NOM_______________<br>LIGNE NO. ☐<br><br>NOM_______________<br>LIGNE NO. ☐	NOM_______________<br>LIGNE NO. ☐<br><br>NOM_______________<br>LIGNE NO. ☐<br><br>NOM_______________<br>LIGNE NO. ☐<br><br>NOM_______________<br>LIGNE NO. ☐<br><br>NOM_______________<br>LIGNE NO. ☐

MOUSTIQUAIRES OBTENU DEPUIS DÉPLACEMENT QUE VOUS N'AVEZ PLUS

LIGNE NO.	QUESTIONS	MOUSTIQUAIRE #1	MOUSTIQUAIRE #2
31	QUAND AVEZ-VOUS RECU LA MOUSTIQUAIRE?	DEPUIS ___ MOIS ☐	DEPUIS ___ MOIS ☐
32	EST-CE QUE VOUS AVEZ REÇU LA MOUSTIQUAIRE PAR UNE CAMPAGNE DE DISTRIBUTION QUAND VOUS ETTE ARRIVÉE AU CAMP?	OUI ☐<br>NO ☐<br>NE SAIT PAS ☐	OUI ☐<br>NO ☐<br>NE SAIT PAS ☐
33	EST-CE QUE VOUS AVEZ REÇU LA MOUSTIQUAIRE OU PENDANT UNE VISITE PRÉNATALS?	OUI ☐<br>NON ☐<br>NE SAIT PAS ☐	OUI ☐<br>NON ☐<br>NE SAIT PAS ☐
34	OU AVEZ-VOUS REÇU LA MOUSTIQUAIRE?	HOPITAL/CENTRE DE SANTÉ/POSTE DE SANTÉ PUBLIC ☐<br>HOPITAL/CLINIQUE PRIVE ☐<br>PHARMACIE ☐<br>MARCHÉ ☐<br>AGENT DE SANTÉ COMMUNAUTAIRE ☐<br>INSTITUT RELIGIEUX ☐<br>ÉCOLE ☐<br>AUTRE (précisez) ☐<br>_________________<br>NE SAIT PAS ☐	HOPITAL/CENTRE DE SANTÉ/POSTE DE SANTÉ PUBLIC ☐<br>HOPITAL/CLINIQUE PRIVE ☐<br>PHARMACIE ☐<br>MARCHÉ ☐<br>AGENT DE SANTÉ COMMUNAUTAIRE ☐<br>INSTITUT RELIGIEUX ☐<br>ÉCOLE ☐<br>AUTRE (précisez) ☐<br>_________________<br>NE SAIT PAS ☐

35	QU'EST CE QUI EST ARRIVÉ AVEC LA MOUSTIQUAIRE ?	VENDU ☐<br>ÉCHANGÉ ☐<br>DONNÉ COMME CADEAU ☐<br>A LA POUBELLE ☐<br>UTILIS POUR AUTRE CHOSE (ex : filet de pèche, etc.) (précisez) ☐<br>________________	VENDU ☐<br>ÉCHANGÉ ☐<br>DONNÉ COMME CADEAU ☐<br>A LA POUBELLE ☐<br>UTILIS POUR AUTRE CHOSE (ex : filet de pèche, etc.) (précisez) ☐<br>________________
36	QUAND EST-CE QUE VOUS AVEZ VENDU/ÉCHANGÉ/ DONNÉ LA MOUSTIQUAIRE?	EN MOIS ☐	EN MOIS ☐
37	POURQUOI AVEZ-VOUS VENDU/ÉCHANGÉ/DONNÉ VOTRE MOUSTIQUAIRE?<br>ENREGISTREZ TOUT CE QUI EST MENTIONNE<br>INSISTEZ : AUTRES RAISON ?	MOUSTIQUAIRE PAS EFFICACE ☐<br>BESOIN D'ARGENT☐<br>CERTAINS MEMBRES DU MENAGE N'AIME PAS LES MOUSTIQUAIRES ☐<br>TAILLE NON SATISFAISANTE ☐<br>TROP DUR/TROP DE TRAVAIL POUR L'INSTALLATION ☐<br>PEUT PAS CLISSER SOU LE LIT ☐<br>FORME NON SATISFAISANTE ☐<br>MAUVAISE ODEUR ☐<br>CAUSE DES IRRITATIONS/ TOUX ☐<br>REND MALADE ☐<br>DONNE DES NAUSÉES ☐<br>PRODUIT CHIMIQUE DANGEREUX ☐<br>PEUT SUFFOQUER/DIFFICULTÉS RESPIRER ☐<br>CHALEUR ☐<br>SE SALIT VITE ☐<br>OBTIENT DES TROUS TROP VITE ☐<br>AUTRE (précisez) ☐<br>________________<br>NE SAIT PAS ☐	MOUSTIQUAIRE PAS EFFICACE ☐<br>BESOIN D'ARGENT☐<br>CERTAINS MEMBRES DU MENAGE N'AIME PAS LES MOUSTIQUAIRES ☐<br>TAILLE NON SATISFAISANTE ☐<br>TROP DUR/TROP DE TRAVAIL POUR L'INSTALLATION ☐<br>PEUT PAS CLISSER SOU LE LIT ☐<br>FORME NON SATISFAISANTE ☐<br>MAUVAISE ODEUR ☐<br>CAUSE DES IRRITATIONS/ TOUX ☐<br>REND MALADE ☐<br>DONNE DES NAUSÉES ☐<br>PRODUIT CHIMIQUE DANGEREUX ☐<br>PEUT SUFFOQUER/DIFFICULTÉS RESPIRER ☐<br>CHALEUR ☐<br>SE SALIT VITE ☐<br>OBTIENT DES TROUS TROP VITE ☐<br>AUTRE (précisez) ☐<br>________________<br>NE SAIT PAS ☐

38	SI LA MOUSTIQUAIRE A ÉTÉ VENDU/ÉCHANGÉ, OU AVEZ-VOUS VENDU/ÉCHANGÉ LA MOUSTIQUAIRE?	VILLAGE ☐<br>MARCHÉ ☐<br>CAMP ☐	VILLAGE ☐<br>MARCHÉ ☐<br>CAMP ☐
39	QUI A ACHETÉ/ACCEPTÉ EN ÉCHANGE LA MOUSTIQUAIRE?	VILLAGEOIS ☐<br>PERSONNES DÉPLACÉES ☐<br>TRAVAILLEUR ONG ☐<br>EMPLOYÉ DU GOUVERNEMENT ☐	VILLAGEOIS ☐<br>PERSONNES DÉPLACÉES ☐<br>TRAVAILLEUR ONG ☐<br>EMPLOYÉ DU GOUVERNEMENT ☐
40	SI LA MOUSTIQUAIRE A ÉTÉ VENDU, A QUEL PRIX AVEZ-VOUS VENDU VOTRE MOUSTIQUAIRE?	PRIX ☐	PRIX ☐

CHARACTÉRISTIQUES SOCIODÉMOGRAPHIQUES

LIGNE NO.	QUESTIONS	RÉSPONSES
41	Est-ce-que le ménage possède un des éléments suivants?	
	ELECTRICITÉ	OUI ☐<br>NON ☐
	RÉFRIGÉRATEUR	OUI ☐<br>NON ☐
	TÉLÉVISION	OUI ☐<br>NON ☐
	RADIO	OUI ☐<br>NON ☐
42	Y-a-t-il un membre du ménage qui possède un des éléments suivants ?	
	TÉLÉPHONE PORTABLE	OUI ☐<br>NON ☐
	VÉLO	OUI ☐<br>NON ☐
	VEHICULE	OUI ☐<br>NON ☐
	MONTRE	OUI ☐<br>NON ☐
43	Est-ce-que le ménage possède un des suivants?	
	VOLAILLE (Poulets/Canards/etc)	OUI ☐<br>NON ☐
	CHEVRES	OUI ☐<br>NON ☐
	VACHES	OUI ☐<br>NON ☐
44	COMBIEN DE TENTES/ABRIS AVEZ VOUS POUR VOTRE MÉNAGE ?	☐

45	OBSERVEZ LE MATÉRIEL DE REVÊTEMENT DU SOL.<br><br>NOTEZ VOS OBSERVATIONS.	SOL NATUREL<br>TERRE/SABLE ☐<br>BOUSE ☐<br><br>SOL RUDIMENTAIRE<br>PLANCHES EN BOIS ☐<br><br>SOL FINI<br>BOIS POLI ☐<br>CIMENT ☐<br>CARRELAGE ☐<br><br>AUTRE ☐
46	OBSERVEZ LE MATÉRIEL DES TOIT OF THE DWELLING.<br><br>NOTEZ VOS OBSERVATIONS.	TOIT NATUREL<br>PAS DE TOIT ☐<br>PALME/CHAUME ☐<br><br>TOIT RUDIMENTAIRE<br>TAPIS ☐<br>PLANCHES EN BOIS ☐<br>TOILE/PLASTIQUE ☐<br>BÂCHE ☐<br><br>TOIT FINI<br>MÉTAL ☐<br>BOIS ☐<br>CÉRAMIQUE ☐<br>CIMENT ☐<br>BARDEAUX ☐<br><br>AUTRE ☐
47	OBSERVEZ LE MATÉRIEL DES MURS EXTÉRIEURS.<br><br>NOTEZ VOS OBSERVATIONS.	MURS NATUREL<br>PAS DE MURS ☐<br>TERRE ☐<br><br>MURS RUDIMENTAIRE<br>PIERRES AVEC BOUE ☐<br>PLANCHES EN BOIS ☐<br>CARTON ☐<br>BÂCHE ☐<br>PALME/BRANCHES ☐<br><br>MURS FINI<br>BRIQUES ☐<br>BLOCS DE CIMENT ☐<br>CIMENT ☐<br><br>AUTRE ☐

AUTRE QUESTIONS :

48) DEPUIS COMBIEN DE TEMPS EST-CE QUE VOTRE MÉNAGE A ÉTÉ DÉPLACÉ? ___________________________________

49) DEPUIS COMBIEN DE TEMPS EST-CE QUE VOTRE MÉNAGE A VECU DANS CE CAMP? ____________________________

OBSERVATIONS DE L'INTERVIEWEUR

A REMPLIR APRES LE SONDAGE

COMMENTAIRES SUR LE SONDAGE:

COMMENTAIRES SUR DES QUESTIONS SPECIFIQUE:

AUTRE COMMENTAIRES:
