# Supplementary material for: Use and disuse of malaria bed nets in an internally displaced persons camp in the Democratic Republic of the Congo: A mixed-methods study
Source: PLoS One. 2017 Sep 26;12(9):e0185290. doi: 10.1371/journal.pone.0185290 (PMC5614551; doi:10.1371/journal.pone.0185290)
Supplement: S3 Table — Community health workers visited a random sample of 100 households in Birambizo IDP camp, asking these questionnaire questions and recording participant answers. The questionnaire was originally created in French and has been translated to English. (PDF) [file pone.0185290.s004.pdf]

MALARIA INDICATOR SURVEY

DATE____ INTERVIEWER_____________________________________

LOCATION____________________________________ HOUSEHOLD REGISTRATION NUMBER:_________________

HOUSEHOLD COMPOSITION

LINE NO.	HOUSEHOLD MEMBERS AND VISITORS	RELATION-SHIP TO HEAD OF HOUSE	SEX	AGE	RESIDENCE		BED NET	MALARIA		VIOLENCE
1	2	3	4	5	6	7	8	9	10	11
	Please provide the names of the individuals who normally live in this household and the visitors who stayed here last night, starting with the head of the household.<br>AFTER LISTING THE NAMES, REGISTERING THE RELATIONSHIP TO THE HEAD OF HOUSEHOLD, AND THE SEX OF EACH PERSON, ASK QUESTIONS 2A-2C TO MAKE SURE THE LIST IS COMPLETE.<br>NEXT, ASK QUESTIONS 5-11 FOR EACH PERSON LISTED.	What is the relationship of (NAME) to the head of the household?<br>SEE CODES BELOW	Is (NAME) male or female?	How old is (NAME)?<br>IN YEARS	Does (NAME) usually sleep in this tent?	Did (NAME) sleep in this tent last night?	Did (NAME) sleep under a bed net last night?	Has (NAME) had any of the following symptoms today?	RDT result?	Has (NAME) been the victim of one or more of the following since their displacement?
01			M ☐<br>F ☐		YES ☐<br>NO ☐	YES ☐<br>NO ☐	YES ☐<br>NO ☐	HEADACHE ☐<br>MYALGIA ☐<br>FEVER ☐	RDT +☐<br>RDT - ☐	THEFT ☐<br>AGRESSION ☐<br>SEXUAL AGRESSION/ RAPE ☐<br>STABBING ☐<br>SHOOTING ☐
02			M ☐<br>F ☐		YES ☐<br>NO ☐	YES ☐<br>NO ☐	YES ☐<br>NO ☐	HEADACHE ☐<br>MYALGIA ☐<br>FEVER ☐	RDT +☐<br>RDT - ☐	THEFT ☐<br>AGRESSION ☐<br>SEXUAL AGRESSION/ RAPE ☐<br>STABBING ☐<br>SHOOTING ☐
03			M ☐<br>F ☐		YES ☐<br>NO ☐	YES ☐<br>NO ☐	YES ☐<br>NO ☐	HEADACHE ☐<br>MYALGIA ☐<br>FEVER ☐	RDT +☐<br>RDT - ☐	THEFT ☐<br>AGRESSION ☐<br>SEXUAL AGRESSION/ RAPE ☐<br>STABBING ☐<br>SHOOTING ☐
04			M ☐<br>F ☐		YES ☐<br>NO ☐	YES ☐<br>NO ☐	YES ☐<br>NO ☐	HEADACHE ☐<br>MYALGIA ☐<br>FEVER ☐	RDT +☐<br>RDT - ☐	THEFT ☐<br>AGRESSION ☐<br>SEXUAL AGRESSION/ RAPE ☐<br>STABBING ☐<br>SHOOTING ☐

05			M ☐<br>F ☐		YES ☐<br>NO ☐	YES ☐<br>NO ☐	YES ☐<br>NO ☐	HEADACHE ☐<br>MYALGIA ☐<br>FEVER ☐	RDT +☐<br>RDT - ☐	THEFT ☐<br>AGRESSION ☐<br>SEXUAL AGRESSION/<br>RAPE ☐<br>STABBING ☐<br>SHOOTING ☐
06			M ☐<br>F ☐		YES ☐<br>NO ☐	YES ☐<br>NO ☐	YES ☐<br>NO ☐	HEADACHE ☐<br>MYALGIA ☐<br>FEVER ☐	RDT +☐<br>RDT - ☐	THEFT ☐<br>AGRESSION ☐<br>SEXUAL AGRESSION/<br>RAPE ☐<br>STABBING ☐<br>SHOOTING ☐
07			M ☐<br>F ☐		YES ☐<br>NO ☐	YES ☐<br>NO ☐	YES ☐<br>NO ☐	HEADACHE ☐<br>MYALGIA ☐<br>FEVER ☐	RDT +☐<br>RDT - ☐	THEFT ☐<br>AGRESSION ☐<br>SEXUAL AGRESSION/<br>RAPE ☐<br>STABBING ☐<br>SHOOTING ☐
08			M ☐<br>F ☐		YES ☐<br>NO ☐	YES ☐<br>NO ☐	YES ☐<br>NO ☐	HEADACHE ☐<br>MYALGIA ☐<br>FEVER ☐	RDT +☐<br>RDT - ☐	THEFT ☐<br>AGRESSION ☐<br>SEXUAL AGRESSION/<br>RAPE ☐<br>STABBING ☐<br>SHOOTING ☐
09			M ☐<br>F ☐		YES ☐<br>NO ☐	YES ☐<br>NO ☐	YES ☐<br>NO ☐	HEADACHE ☐<br>MYALGIA ☐<br>FEVER ☐	RDT +☐<br>RDT - ☐	THEFT ☐<br>AGRESSION ☐<br>SEXUAL AGRESSION/<br>RAPE ☐<br>STABBING ☐<br>SHOOTING ☐
10			M ☐<br>F ☐		YES ☐<br>NO ☐	YES ☐<br>NO ☐	YES ☐<br>NO ☐	HEADACHE ☐<br>MYALGIA ☐<br>FEVER ☐	RDT +☐<br>RDT - ☐	THEFT ☐<br>AGRESSION ☐<br>SEXUAL AGRESSION/<br>RAPE ☐<br>STABBING ☐<br>SHOOTING ☐

2A) Just to be sure that I have a complete list: are there any other adults of children that we haven't listed? (If yes, add them to the table)
2B) Are there others who maybe aren't family members who are friends or friends of neighbors who normally live here? (If yes, add them to the table)
2C) Are there guests or temporary visitors or other people who slept here last night and who aren't yet listed? (If yes, add them to the table)

CODES FOR Q.3: RELATIONSHIP TO THE HEAD OF HOUSEHOLD

01 = HEAD OF HOUSEHOLD
02 = SPOUSE
03 = SON OR DAUGHTER
04 = SON OR DAUGHTER IN-LAW
05= STEP SON OR DAUGHTER
06 = MOTHER/FATHER
07 = PARENT IN-LAW
08 = BROTHER OR SISTER
09 = OTHER PARENT
10 = ADOPTED/ FOSTERED CHILD
11 = SPOUSE'S CHILDREN
12 = OTHER RELATIVE
13 = NO RELATIONSHIP
98 = DO NOT KNOW

CHILDREN UNDER FIVE

LINE NO.	NAMES OF CHILDREN UNDER 5 YEARS	FEVER		MEDICAL		MALARIA		
1	2	3	4	5	6	7	8	9
11	Please list the names of the children under 5 years of age and the line number from the household composition table.	Has the child had a fever in the past month?	If yes, did you seek medical advice or treatment at the medical centre?	Did the child have malaria?	If yes, was the diagnosis confirmed by RDT or microscopy?	Was antimalarial treatment administered?	What is the level of education of this child's mother?	Is this child's mother literate?
12	NAME________________ LINE NO. ☐	YES ☐ NO ☐	YES ☐ NO ☐	YES ☐ NO ☐	YES ☐ NO ☐	YES ☐ NO ☐	NONE ☐ PRIMARY ☐ SECONDARY ☐ POST SECONDARY ☐	YES ☐ NO ☐
13	NAME________________ LINE NO. ☐	YES ☐ NO ☐	YES ☐ NO ☐	YES ☐ NO ☐	YES ☐ NO ☐	YES ☐ NO ☐	NONE ☐ PRIMARY ☐ SECONDARY ☐ POST SECONDARY ☐	YES ☐ NO ☐
14	NAME________________ LINE NO. ☐	YES ☐ NO ☐	YES ☐ NO ☐	YES ☐ NO ☐	YES ☐ NO ☐	YES ☐ NO ☐	NONE ☐ PRIMARY ☐ SECONDARY ☐ POST SECONDARY ☐	YES ☐ NO ☐
15	NAME________________ LINE NO. ☐	YES ☐ NO ☐	YES ☐ NO ☐	YES ☐ NO ☐	YES ☐ NO ☐	YES ☐ NO ☐	NONE ☐ PRIMARY ☐ SECONDARY ☐ POST SECONDARY ☐	YES ☐ NO ☐
16	NAME________________ LINE NO. ☐	YES ☐ NO ☐	YES ☐ NO ☐	YES ☐ NO ☐	YES ☐ NO ☐	YES ☐ NO ☐	NONE ☐ PRIMARY ☐ SECONDARY ☐ POST SECONDARY ☐	YES ☐ NO ☐

BED NETS

LINE NO.		BED NET #1	BED NET #2	BED NET #3
17	ASK TO SEE ALL BED NETS IN THE HOUSEHOLD<br><br>IF THERE ARE MORE THAN 3 NETS, USE MORE SURVEYS	OBSERVED ☐<br>NOT OBSERVED ☐	OBSERVED ☐<br>NOT OBSERVED ☐	OBSERVED ☐<br>NOT OBSERVED ☐
18	HOW LONG HAS YOUR HOUSEHOLD HAD THIS NET?	IN MONTHS ☐	IN MONTHS ☐	IN MONTHS ☐
19	DID YOU RECEIVE THE BED NET DURING A DISTRIBUTION CAMPAIGN WHEN YOU ARRIVED AT THE CAMP?	YES ☐<br>NO ☐<br>DO NOT KNOW ☐	YES ☐<br>NO ☐<br>DO NOT KNOW ☐	YES ☐<br>NO ☐<br>DO NOT KNOW ☐
20	DID YOU RECEIVE THE BED NET DURING A PRENATAL VISIT?	YES ☐<br>NO ☐<br>DO NOT KNOW ☐	YES ☐<br>NO ☐<br>DO NOT KNOW ☐	YES ☐<br>NO ☐<br>DO NOT KNOW ☐
21	WHERE DID YOU RECEIVE THE BED NET?	NGO DISTRIBUTION ☐<br><br>HOSPITAL/HEALTH CENTRE/PUBLIC HEALTH FACILITY ☐<br><br>PRIVATE HOSPITAL/CLINIC ☐<br><br>PHARMACY ☐<br><br>MARKET ☐<br><br>COMMUNITY HEALTH WORKER ☐<br><br>RELIGIOUS INSTITUTION ☐<br><br>SCHOOL ☐<br><br>OTHER (specify) ☐<br>________________<br><br>DO NOT KNOW ☐	NGO DISTRIBUTION ☐<br><br>HOSPITAL/HEALTH CENTRE/PUBLIC HEALTH FACILITY ☐<br><br>PRIVATE HOSPITAL/CLINIC ☐<br><br>PHARMACY ☐<br><br>MARKET ☐<br><br>COMMUNITY HEALTH WORKER ☐<br><br>RELIGIOUS INSTITUTION ☐<br><br>SCHOOL ☐<br><br>OTHER (specify) ☐<br>________________<br><br>DO NOT KNOW ☐	NGO DISTRIBUTION ☐<br><br>HOSPITAL/HEALTH CENTRE/PUBLIC HEALTH FACILITY ☐<br><br>PRIVATE HOSPITAL/CLINIC ☐<br><br>PHARMACY ☐<br><br>MARKET ☐<br><br>COMMUNITY HEALTH WORKER ☐<br><br>RELIGIOUS INSTITUTION ☐<br><br>SCHOOL ☐<br><br>OTHER (specify) ☐<br>________________<br><br>DO NOT KNOW ☐
22	IS THE BED NET USED BY THE HOUSEHOLD?<br><br>IF YES, GO TO Q24, IF NO, ASK Q23 AND PROCEED TO Q31.	YES ☐<br>NO ☐<br>DO NOT KNOW ☐	YES ☐<br>NO ☐<br>DO NOT KNOW ☐	YES ☐<br>NO ☐<br>DO NOT KNOW ☐

23	WHY DOESN'T YOUR HOUSEHOLD USE THE BED NET?<br><br>SELECT ALL THAT WERE MENTIONED<br><br>INSIST: ARE THERE ANY OTHER REASONS?	BED NET NOT EFFICACIOUS ☐<br>NEEDED MONEY ☐<br>SOME MEMBERS OF THE HOUSEHOLD DON'T LIKE IT ☐<br>SIZE IS UNSATISFACTORY ☐<br>INSTALLATION DIFFICULT ☐<br>CAN'T TUCK UNDER A MATTRESS ☐<br>SHAPE IS UNSATISFACTORY ☐<br>SMELLS BADLY ☐<br>CAUSES IRRITATIONS/COUGHING ☐<br>CAUSES SICKNESS ☐<br>CAUSES NAUSEA ☐<br>PRODUCES DANGEROUS CHEMICAL ☐<br>CAN SUFFOCATE/CAUSES DIFFICULTY BREATHING ☐<br>TOO HOT ☐<br>GETS DIRTY TOO QUICKLY ☐<br>GETS HOLES TOO QUICKLY ☐<br>OTHER (specify) ☐<br>________________<br>DO NOT KNOW ☐	BED NET NOT EFFICACIOUS ☐<br>NEEDED MONEY ☐<br>SOME MEMBERS OF THE HOUSEHOLD DON'T LIKE IT ☐<br>SIZE IS UNSATISFACTORY ☐<br>INSTALLATION DIFFICULT ☐<br>CAN'T TUCK UNDER A MATTRESS ☐<br>SHAPE IS UNSATISFACTORY ☐<br>SMELLS BADLY ☐<br>CAUSES IRRITATIONS/COUGHING ☐<br>CAUSES SICKNESS ☐<br>CAUSES NAUSEA ☐<br>PRODUCES DANGEROUS CHEMICAL ☐<br>CAN SUFFOCATE/CAUSES DIFFICULTY BREATHING ☐<br>TOO HOT ☐<br>GETS DIRTY TOO QUICKLY ☐<br>GETS HOLES TOO QUICKLY ☐<br>OTHER (specify) ☐<br>________________<br>DO NOT KNOW ☐	BED NET NOT EFFICACIOUS ☐<br>NEEDED MONEY ☐<br>SOME MEMBERS OF THE HOUSEHOLD DON'T LIKE IT ☐<br>SIZE IS UNSATISFACTORY ☐<br>INSTALLATION DIFFICULT ☐<br>CAN'T TUCK UNDER A MATTRESS ☐<br>SHAPE IS UNSATISFACTORY ☐<br>SMELLS BADLY ☐<br>CAUSES IRRITATIONS/COUGHING ☐<br>CAUSES SICKNESS ☐<br>CAUSES NAUSEA ☐<br>PRODUCES DANGEROUS CHEMICAL ☐<br>CAN SUFFOCATE/CAUSES DIFFICULTY BREATHING ☐<br>TOO HOT ☐<br>GETS DIRTY TOO QUICKLY ☐<br>GETS HOLES TOO QUICKLY ☐<br>OTHER (specify) ☐<br>________________<br>DO NOT KNOW ☐
24	SINCE YOU HAVE HAD THE BED NET, HAS IT DEVELOPED ANY HOLES?	YES ☐<br>NO ☐<br>DO NOT KNOW ☐	YES ☐<br>NO ☐<br>DO NOT KNOW ☐	YES ☐<br>NO ☐<br>DO NOT KNOW ☐
25	IF YES, HOW MANY HOLES DOES IT HAVE?	MANY ☐<br>FEW ☐	MANY ☐<br>FEW ☐	MANY ☐<br>FEW ☐
26	DO YOU WASH THE BED NET?	YES ☐<br>NO ☐<br>DO NOT KNOW ☐	YES ☐<br>NO ☐<br>DO NOT KNOW ☐	YES ☐<br>NO ☐<br>DO NOT KNOW ☐
27	IF YES, HOW MANY DAYS HAS IT BEEN SINCE IT WAS LAST WASHED?	IN DAYS ☐	IN DAYS ☐	IN DAYS ☐
28	SINCE YOU HAVE HAD THE BED NET, HAS IT BEEN TREATED WITH	YES ☐<br>NO ☐<br>DO NOT KNOW ☐	YES ☐<br>NO ☐<br>DO NOT KNOW ☐	YES ☐<br>NO ☐<br>DO NOT KNOW ☐

	AN INSECTICIDE TO KILL OR DETER MOSQUITOES?			
29	LAST NIGHT, DID SOMEONE SLEEP UNDER THIS BED NET?	YES ☐<br>NO ☐<br>DO NOT KNOW ☐	YES ☐<br>NO ☐<br>DO NOT KNOW ☐	YES ☐<br>NO ☐<br>DO NOT KNOW ☐
30	IF YES, WHO SLEPT UNDER THIS BED NET LAST NIGHT?<br><br>LIST ALL NAMES AND LINE NUMBERS FROM THE HOUSEHOLD COMPOSITION TABLE.	NAME________________<br>LINE NO. ☐<br><br>NAME________________<br>LINE NO. ☐<br><br>NAME________________<br>LINE NO. ☐<br><br>NAME________________<br>LINE NO. ☐<br><br>NAME________________<br>LINE NO. ☐	NAME________________<br>LINE NO. ☐<br><br>NAME________________<br>LINE NO. ☐<br><br>NAME________________<br>LINE NO. ☐<br><br>NAME________________<br>LINE NO. ☐<br><br>NAME________________<br>LINE NO. ☐	NAME________________<br>LINE NO. ☐<br><br>NAME________________<br>LINE NO. ☐<br><br>NAME________________<br>LINE NO. ☐<br><br>NAME________________<br>LINE NO. ☐<br><br>NAME________________<br>LINE NO. ☐

BED NETS RECEIVED SINCE DISPLACEMENT THAT YOU NO LONGER HAVE

LINE NO.	QUESTIONS	BED NET #1	BED NET #2
31	HOW LONG HAS YOUR HOUSEHOLD HAD THIS NET?	IN MONTHS ☐	IN MONTHS ☐
32	DID YOU RECEIVE THE BED NET DURING A DISTRIBUTION CAMPAIGN WHEN YOU ARRIVED AT THE CAMP?	YES ☐<br>NO ☐<br>DO NOT KNOW ☐	YES ☐<br>NO ☐<br>DO NOT KNOW ☐
33	DID YOU RECEIVE THE BED NET DURING A PRENATAL VISIT?	YES ☐<br>NO ☐<br>DO NOT KNOW ☐	YES ☐<br>NO ☐<br>DO NOT KNOW ☐
34	WHERE DID YOU RECEIVE THE BED NET?	NGO DISTRIBUTION ☐<br><br>HOSPITAL/MEDICAL CENTRE/PUBLIC HEALTH FACILITY ☐<br><br>PRIVATE HOSPITAL/CLINIC ☐<br><br>PHARMACY ☐<br><br>MARKET ☐<br><br>COMMUNITY HEALTH WORKER ☐<br><br>RELIGIOUS INSTITUTION ☐<br><br>SCHOOL ☐<br><br>OTHER (specify) ☐	NGO DISTRIBUTION ☐<br><br>HOSPITAL/MEDICAL CENTRE/PUBLIC HEALTH FACILITY ☐<br><br>PRIVATE HOSPITAL/CLINIC ☐<br><br>PHARMACY ☐<br><br>MARKET ☐<br><br>COMMUNITY HEALTH WORKER ☐<br><br>RELIGIOUS INSTITUTION ☐<br><br>SCHOOL ☐<br><br>OTHER (specify) ☐

		______________<br>DO NOT KNOW ☐	______________<br>DO NOT KNOW ☐
35	WHAT HAPPENED TO THE BED NET?	SOLD ☐<br>EXCHANGED ☐<br>GAVE AWAY AS A GIFT ☐<br>THREW IT OUT ☐<br>USED IT FOR OTHER THINGS (eg: fish net, etc.) (specify) ☐<br>________________	SOLD ☐<br>EXCHANGED ☐<br>GAVE AWAY AS A GIFT ☐<br>THREW IT OUT ☐<br>USED IT FOR OTHER THINGS (eg: fish net, etc.) (specify) ☐<br>________________
36	WHEN DID YOU SELL/EXCHANGE/ GIVE AWAY THE BED NET?	IN MONTHS ☐	IN MONTHS ☐
37	WHY DID YOU SELL/EXCHANGE/ GIVE AWAY THE BED NET?<br><br>CHECK ALL THAT ARE MENTIONED<br><br>INSIST: ARE THERE ANY OTHER REASONS?	BED NET NOT EFFICACIOUS ☐<br>NEEDED MONEY ☐<br>SOME MEMBERS OF THE HOUSEHOLD DON'T LIKE IT ☐<br>SIZE IS UNSATISFACTORY ☐<br>INSTALLATION DIFFICULT ☐<br>CAN'T TUCK UNDER A MATTRESS ☐<br>SHAPE IS UNSATISFACTORY ☐<br>SMELLS BADLY ☐<br>CAUSES IRRITATIONS/COUGHING ☐<br>CAUSES SICKNESS ☐<br>CAUSES NAUSEA ☐<br>PRODUCES DANGEROUS CHEMICAL ☐<br>CAN SUFFOCATE/CAUSES DIFFICULTY BREATHING ☐<br>TOO HOT ☐<br>GETS DIRTY TOO QUICKLY ☐<br>GETS HOLES TOO QUICKLY ☐<br>OTHER (specify) ☐<br>________________<br>DO NOT KNOW ☐	BED NET NOT EFFICACIOUS ☐<br>NEEDED MONEY ☐<br>SOME MEMBERS OF THE HOUSEHOLD DON'T LIKE IT ☐<br>SIZE IS UNSATISFACTORY ☐<br>INSTALLATION DIFFICULT ☐<br>CAN'T TUCK UNDER A MATTRESS ☐<br>SHAPE IS UNSATISFACTORY ☐<br>SMELLS BADLY ☐<br>CAUSES IRRITATIONS/COUGHING ☐<br>CAUSES SICKNESS ☐<br>CAUSES NAUSEA ☐<br>PRODUCES DANGEROUS CHEMICAL ☐<br>CAN SUFFOCATE/CAUSES DIFFICULTY BREATHING ☐<br>TOO HOT ☐<br>GETS DIRTY TOO QUICKLY ☐<br>GETS HOLES TOO QUICKLY ☐<br>OTHER (specify) ☐<br>________________<br>DO NOT KNOW ☐

38	IF THE BED NET WAS SOLD/EXCHANGED, WHERE DID YOU SELL/EXCHANGE THE BED NET?	MARKET ☐<br>CAMP ☐	MARKET ☐<br>CAMP ☐
39	WHO BOUGHT/ACCEPTED THE NED NET?	VILLAGER ☐<br>DISPLACED PERSON ☐<br>NGO WORKER ☐<br>GOVERNMENT EMPLOYEE ☐	VILLAGER ☐<br>DISPLACED PERSON ☐<br>NGO WORKER ☐<br>GOVERNMENT EMPLOYEE ☐
40	IF THE BED NET WAS SOLD, WHAT PRICE DID YOU SELL IT FOR?	PRICE ☐	PRICE ☐

SOCIODEMOGRAPHICS

LINE NO.	QUESTIONS	ANSWERS
41	Does the household have any of the following?<br><br>ELECTRICITY<br><br>REFRIGERATOR<br><br>TELEVISION<br><br>RADIO	 <br>YES ☐<br>NO ☐<br>YES ☐<br>NO ☐<br>YES ☐<br>NO ☐<br>YES ☐<br>NO ☐
42	Does any member of the household have one of the following?<br><br>MOBILE PHONE<br><br>BICYCLE<br><br>VEHICLE<br><br>WATCH	 <br>YES ☐<br>NO ☐<br>YES ☐<br>NO ☐<br>YES ☐<br>NO ☐<br>YES ☐<br>NO ☐
43	Does the household own any of the following?<br><br>POULTRY (Chickens/Ducks/etc)<br><br>GOAT(S)<br><br>COW(S)	 <br>YES ☐<br>NO ☐<br>YES ☐<br>NO ☐<br>YES ☐<br>NO ☐
44	HOW MANY TENTS IS THE HOUSEHOLD MADE UP OF	☐

45	OBSERVE THE MATERIAL OF THE TENT FLOOR.<br><br>NOTE YOUR OBSERVATIONS.	NATURAL FLOORING<br>EARTH/SAND ☐<br>MUD ☐<br><br>RUDIMENTARY FLOORING<br>WOOD BOARDS ☐<br>TARPAULIN ☐<br><br>FINISHED FLOORING<br>FINISHED WOOD ☐<br>CEMENT ☐<br>TILES ☐<br><br>OTHER ☐
46	OBSERVE THE MATERIAL OF THE ROOF.<br><br>NOTE YOUR OBSERVATIONS.	NATURAL ROOFING<br>PAS DE TOIT ☐<br>STRAW/PALM LEAVES ☐<br><br>RUDIMENTARY ROOFING<br>RUG ☐<br>WOOD BOARDS ☐<br>TARPAULIN ☐<br><br>FINISHED ROOFING<br>METAL ☐<br>WOOD ☐<br>CERAMIC ☐<br>CEMENT ☐<br>SHINGLES ☐<br><br>OTHER ☐
47	OBSERVE THE MATERIAL OF THE WALLS.<br><br>NOTE YOUR OBSERVATIONS.	NATURAL<br>NO WALLS ☐<br>EARTH ☐<br><br>RUDIMENTARY WALLS<br>ROCKS AND MUD ☐<br>WOOD BOARDS ☐<br>CARDBOARD ☐<br>TARPAULIN ☐<br>PALMS/BRANCHES ☐<br><br>FINISHED WALLS<br>BRICKS ☐<br>CEMENT BLOCKS ☐<br>CEMENT ☐<br><br>OTHER ☐

OTHER QUESTIONS:

48) HOW LONG HAS YOUR HOUSEHOLD BEEN DISPLACED (MONTHS)? ___________________________

49) HOW LONG HAS YOUR HOSUEHOLD LIVED IN THIS CAMP (MONTHS)?__________________________

INTERVIEWER OBSERVATIONS

TO BE FILLED OUT AFTER SURVEY COMPLETION

COMMENTS ABOUT THE SURVEY:

COMMENTS ABOUT SPECIFIC QUESTIONS:

OTHER COMMENTS:
